# Lymphatic Mapping in Colon Cancer Depending on Injection Time and Tracing Agent: A Systematic Review and Meta-Analysis of Prospective Designed Studies

**DOI:** 10.3390/cancers15123196

**Published:** 2023-06-15

**Authors:** Katharina Lucas, Nathaniel Melling, Anastasios D. Giannou, Matthias Reeh, Oliver Mann, Thilo Hackert, Jakob R. Izbicki, Daniel Perez, Julia K. Grass

**Affiliations:** 1 Department of General, Visceral and Thoracic Surgery, University Medical Center Hamburg-Eppendorf, Martinistraße 52, 20246 Hamburg, Germany; katharina.lucas@uni-wh.de (K.L.); n.melling@uke.de (N.M.); a.giannou@uke.de (A.D.G.); m.reeh@uke.de (M.R.); o.mann@uke.de (O.M.); t.hackert@uke.de (T.H.); izbicki@uke.de (J.R.I.); d.perez@uke.de (D.P.); 2 Department of Visceral, Thoracic, Vascular Surgery and Angiology, City Hospital Triemli, Birmensdorferstrasse 497, 8063 Zürich, Switzerland; 3 Department of General and Visceral Surgery, Asklepios Hospital Altona, Paul-Ehrlich-Straße 1, 22763 Hamburg, Germany

**Keywords:** colon cancer, lymphatic mapping, lymph node metastases, aberrant drainage, ICG, curative surgery, oncologic surgery, sentinel lymph nodes

## Abstract

**Simple Summary:**

Lymphatic spreading is a main driver of metastasis and, thus, associated death in colon cancer. Therefore, resecting all metastatic lymph nodes is vital for cancer-free survival. Although resection within established resection lines provides a good lymph node yield, aberrant lymphatic drainage pathways may be missed. Lymphatic mapping can compensate for this shortcoming. Different methods for tracing lymphatic drainage exist, such as radiocolloid tracers, ink, and fluorescent tracers. Tracers can be applicated either during surgery or before surgery through colonoscopy, giving the tracer more time to travel through the lymphatic system and highlighting more distant tumor-draining lymph nodes. This review aims to assess which protocol best maps the lymphatic drainage pathway and thus enables an optimized, personalized approach for curative resection.

**Abstract:**

An optimized lymph node yield leads to better survival in colon cancer, but extended lymphadenectomy is not associated with survival benefits. Lymphatic mapping shows several colon cancers feature aberrant drainage pathways inducing local recurrence when not resected. Currently, different protocols exist for lymphatic mapping procedures. This meta-analysis assessed which protocol has the best capacity to detect tumor-draining and possibly metastatic lymph nodes. A systematic review was conducted according to PRISMA guidelines, including prospective trials with in vivo tracer application. The risk of bias was evaluated using the QUADAS-2 tool. Traced lymph nodes, total resected lymph nodes, and aberrant drainage detection rate were analyzed. Fifty-eight studies met the inclusion criteria, of which 42 searched for aberrant drainage. While a preoperative tracer injection significantly increased the traced lymph node rates compared to intraoperative tracing (30.1% (15.4, 47.3) vs. 14.1% (11.9, 16.5), *p* = 0.03), no effect was shown for the tracer used (*p* = 0.740) or the application sites comparing submucosal and subserosal injection (22.9% (14.1, 33.1) vs. 14.3% (12.1, 16.8), *p* = 0.07). Preoperative tracer injection resulted in a significantly higher rate of detected aberrant lymph nodes compared to intraoperative injection (26.3% [95% CI 11.5, 44.0] vs. 2.5% [95% CI 0.8, 4.7], *p* < 0.001). Analyzing 112 individual patient datasets from eight studies revealed a significant impact on aberrant drainage detection for injection timing, favoring preoperative over intraoperative injection (OR 0.050 [95% CI 0.010–0.176], *p* < 0.001) while indocyanine green presented itself as the superior tracer (OR 0.127 [95% CI 0.018–0.528], *p* = 0.012). Optimized lymphatic mapping techniques result in significantly higher detection of aberrant lymphatic drainage patterns and thus enable a personalized approach to reducing local recurrence.

## 1. Introduction

In the curative treatment of colon cancer, long-term survival has increased significantly since optimized lymphadenectomy in complete mesocolic excision (CME) has been carried out [1,2,3]. The CME implies the sharp dissection of the visceral from the retroperitoneal plane, aiming to resect an intact package of the tumor with its main lymphatic drainage, thus maximizing lymph node harvest. However, tumor recurrence and metachronal metastasis affect a substantial proportion of formerly R0 resected patients [4], which raises the need for further improved diagnostics and therapy.

The sentinel technic was developed to highlight a tumor’s first draining lymph node (LN). While precise for other tumor entities, the colonic drainage is not as linear as that of breast cancer or melanoma [5], and skip metastases are frequent findings [6,7]. In CC, sentinel LN mapping has been associated with low sensitivities [8,9,10,11,12], while skip metastases, and metastases in aberrant LNs, can occur. Skip metastases are tumor-positive LNs distal of tumor-negative LNs [13,14], while aberrant LN describes draining LN outside the standard resection margin [7,15,16,17]. Identifying the first few draining LNs via sentinel technique could not produce reliable results in colon cancer with sensitivities of 63–73.7% [8,9,10,11,12] of predicting the nodal status of the disease. Moreover, recent studies detected aberrant drainage patterns outside the known lymphatic drainage routes of CC [18,19]. Possible pathomechanisms leading to this phenomenon include different congenital drainage, lymphangiogenesis, and lymphatic occlusion with consecutive rerouting [20].

Varying protocols have been proposed for lymphatic drainage tracing in CC, which differ in both the tracer used and the application timing and method. Historically common is the intraoperative, subserosal application of ink detected either in pathological assessment or intraoperatively. The same technique was studied with a radiocolloid tracer and the fluorescent tracer indocyanine green (ICG), relying on either a radioactivity detection system or a fluorescent camera system. However, intraoperative tracer application only allows a narrow time frame to visualize lymphatic drainage, but especially aberrant drainage paths with slower lymph flow might be missed. Alternatively, the tracer can be applicated through colonoscopy, injecting the tracer submucosally near the tumor. This can be done directly prior to surgery or a day or more in advance, giving the tracer time to travel through the lymphatic system, staining passed LNs tracer-positive.

Given recurrence rates range between 5 and 10% [21,22] in non-metastatic patients receiving R0 resection in CC and local recurrence originating mostly from lymphatic metastasis [6], there is potential for improvement. Visualizing the individual lymphatic flow might be a key to reducing tumor recurrence and precise staging in CC. This review aims to identify the best protocol to thoroughly visualize a patient’s individual lymphatic drainage pattern in CC to improve oncological outcomes further.

## 2. Materials and Methods

### 2.1. Protocol and Guidance

A systematic review was conducted according to Preferred Reporting Items for Systematic Reviews and Meta-Analyses (PRISMA) guidelines [23]. The protocol was prospectively PROSPERO-registered with the registration ID CRD42021258766 [24].

### 2.2. Data Sources and Search Strategy

A comprehensive database search was performed using Medline and Web of Science, including “forward cited search” [16,25,26], and Embase through OVID. Registers used include Cochrane and PROSPERO. A search string was conducted with the help of an experienced librarian from the University of Hamburg, validated by preliminarily finding already known studies, and translated via SR-accelerators polyglot search [27]. All searches were conducted on 20 July 2021; the original search string can be found in the Supplementary Data (Data S1). A second search using the same search strategies was conducted on 13 February 2023, which led to five newly published studies [28,29,30,31,32]. Reviews [8,9,10,11,12,33,34] concerning similar research topics were manually searched for possible missed publications. One study [35] was found through this process.

### 2.3. Eligibility Criteria

Prospectively designed studies including a minimum of five patients with in vivo tracer application intended for lymphatic mapping in colonic malignancies, followed by oncologic resection in patients aged above 18 years, were eligible. Exclusion criteria consisted of ex vivo mapping, other than standard pTNM staging used, doubled patient data through multiple publications of the same collective, number of nodal positive patients only given including patients upstaged via experimental immunohistochemistry (IHC) analysis of LNs, retrospective study design, other tracing detection used, publications in languages other than English, German, or French, and inclusion of rectal neoplasms without the option for extrapolation of CC patient data, this due to the different lymphatic drainage in the mesorectum and mesocolon. Conference abstracts [36,37] were included if sufficient data were provided.

### 2.4. Study Selection and Data Extraction

Two authors (K.L. and J.-K.G.) individually conducted title, abstract, and full-text screening using Covidence [38]. Conflicts were discussed, and an agreement was reached.

Data extracted included study design, patient number, tracing method and timing, application site, TNM stage, LN tracing procedures, and LN data such as total resected LN and total traced LN, data of aberrant drainage searched and found, number of aberrant LNs, if a change in resection lines was done, and number of changes of resection line. Supplementary Data were considered, and selected authors were contacted for additional information. Individual patient datasets (IPD) were extracted when reported. As tracers ICG, radiocolloids and ink (such as patent blue, isosulfan blue, lymphazurin, methylene blue, carbon particles, or blue dye V) were eligible. Detection methods for aberrant LNs were visibility in the mesentery for ink, usage of an ICG camera before and/or after oncologic resection, and usage of a gamma camera before and/or after the oncologic resection. The preoperative tracer injections took mostly a day, and up to three days prior to surgery place; intraoperative injection was defined as an injection during the surgical procedure after skin incision. Aberrant drainage was defined as LNs outside the standard resection margin. Studies describing a combination of tracers or application methods were included if data for each application or detection method could be extrapolated. In studies assessing upstaging, and thus reporting the pTNM stage with additional IHC staining for detecting micrometastases, which were in some studies considered tumor-positive nodes, the LN status assessed with standard examination techniques was extracted for comparability without bias.

### 2.5. Quality Assessment and Risk of Bias

Quality assessment was done individually by two reviewers (K.L. and J.-K.G.). Discrepancies were discussed, and an agreement was reached. The QUADAS-2 [39] tool with partly reviewed specifically tailored signaling questions. Questions and criteria of quality assessment are described in Table S1. Outcomes of interest were the proportion of traced LNs in all resected LNs according to injection timing, application site and tracer, aberrant nodal positivity, and detection of aberrant lymphatic drainage patterns. Analyzed were overall study results and individual patient data (IPD).

### 2.6. Data Synthesis and Analysis

These meta-analyses were implemented in Stata version 14.2 using the metaprop_one command. When data were available in at least three studies, heterogeneity was assessed by Cochran’s Q test and the I^2^ statistics. Results for each study were pooled using a single-arm meta-analysis of proportions models. Individual study results were analyzed using random-effects models based on the DerSimonian and Laird method, with heterogeneity estimated from the inverse variance model. Wilson score confidence intervals were used, and the Freeman–Tukey double-arcsine transformation was applied to stabilize the variances. To calculate outcomes, assumptions of mean values were made when data were given in median and range. Individual patient data were analyzed using traditional covariate-adjusted linear models and generalized linear models, adjusting for the tracer used, the timing of tracer application, tumor localization, and T-stage. In all instances, a *p*-value < 0.05 was considered statistically significant.

## 3. Results

**Figure 1:**
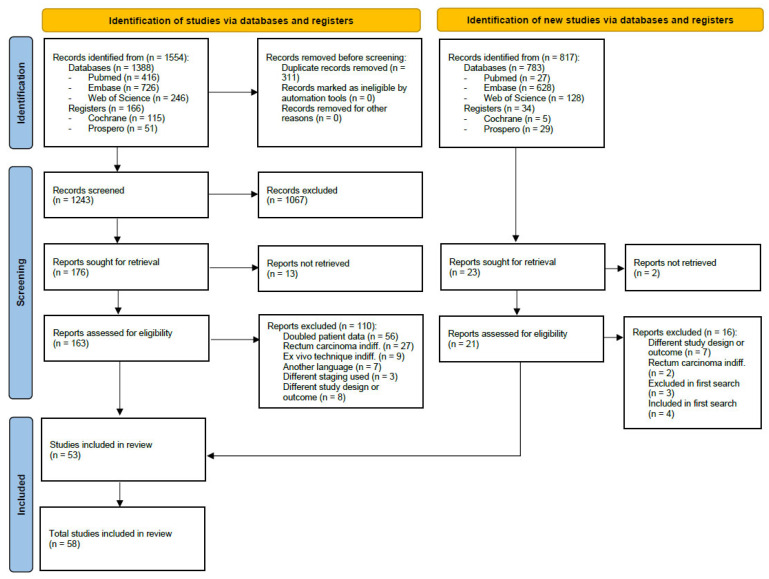
PRISMA 2020 flowchart of study selection process. n—number, indiff. = indifferentiable.

The original search in July 2021 retrieved 1554 results. The selection process is described in Figure 1 [40]. Several studies appearing to meet inclusion criteria were excluded for the following reasons: doubled patient data [7,41,42,43,44,45,46,47,48,49,50,51,52,53,54,55,56,57,58,59,60,61,62,63,64,65,66,67,68,69,70,71,72,73,74,75,76,77,78,79,80,81,82,83,84,85,86,87,88,89,90,91,92,93,94,95], rectum carcinoma not differentiable [33,96,97,98,99,100,101,102,103,104,105,106,107,108,109,110,111,112,113,114,115,116,117,118,119,120,121], another language [122,123,124,125,126,127,128], ex vivo data not differentiable [129,130,131,132,133,134,135,136,137], only upstaged LN data provided [138,139,140], different study design or outcome [141,142,143,144,145,146,147,148]. A second search was conducted on 13 February 2023, to integrate the most recent findings, leading to five furthermore included studies [28,29,30,31,32]. The same search strategy was applied limited to publication dates 2021–2023. Studies were excluded for different designs or outcomes [10,149,150,151,152,153,154], rectum carcinoma included [109,155], in the first search included [15,17,156,157] or excluded [118,121,145] and found again due to overlapping publication dates.

### 3.1. Study Characteristics and Quality Assessment

Fifty-eight studies could be included, analyzing 3393 patients. The majority of studies (89.7%) had a monocentric study design. Nineteen studies used ICG, 30 ink, and five radiocolloid as tracers. Four studies applied a combination of tracers in their cohort. While most studies performed a completely intraoperative tracer application (81.0%), labeling was done preoperatively in nine cohorts and intra- and preoperatively in two studies (Table 1).

Low risk of bias was generally present in the quality assessment using the QUADAS-2 tool. In studies with a high risk of bias for the reference standard, the number of tumor-positive LNs had to be extracted without the experimentally used staging techniques. An overlap of exclusion criteria and the QUADAS-2 suggested index test questions led to included studies showing a low risk of bias in patient selection, index test, and reference standard for the section of applicability concerns. Results can be found in Table 2.

### 3.2. Effectiveness of Lymph Node Mapping

**Figure 2:**
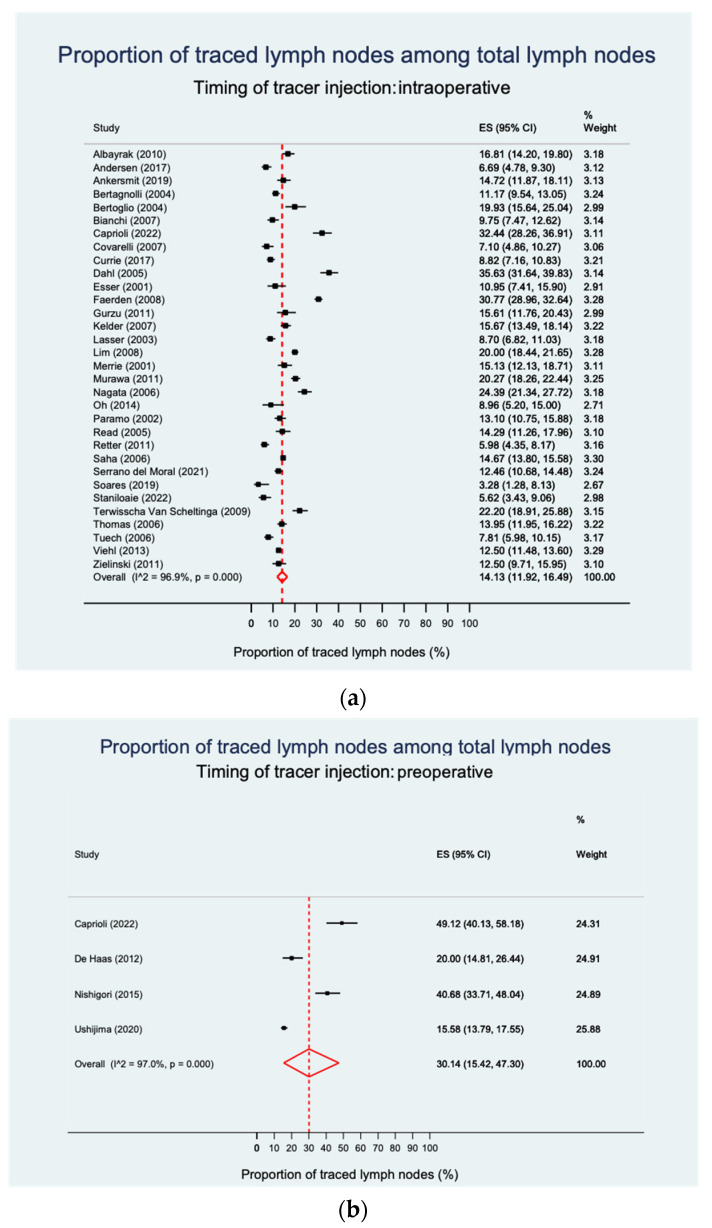
Proportion of tracer-positive LNs among total LNs according to injection timing. (**a**) Intraoperative tracer injection. The pooled estimate of the proportion of traced LNs is 14.1%. (**b**) Preoperative tracer injection. The pooled estimate of the proportion of traced LNs is 30.1%. CI—confidence interval, ES—effect size [12,16,19,25,26,28,32,35,157,158,159,162,163,165,168,169,170,171,174,175,176,178,179,180,181,182,183,187,188,190,192,193,194,195,197].

Thirty-six studies qualified for analysis of LN mapping effectiveness. When studies reported multiple techniques, separate cohorts were considered. After intraoperative tracer injection, proportions of mapped lymph nodes ranged from 3.28 to 35.63% with a pooled rate of 14.1%. In contrast, preoperative LN mapping resulted in a significantly higher pooled rate of 30.1% traced LNs (*p* = 0.030), ranging from 15.58 to 49.12% (Figure 2a,b, and Table S2). Both analyses showed a significantly high level of heterogeneity.

**Figure 3:**
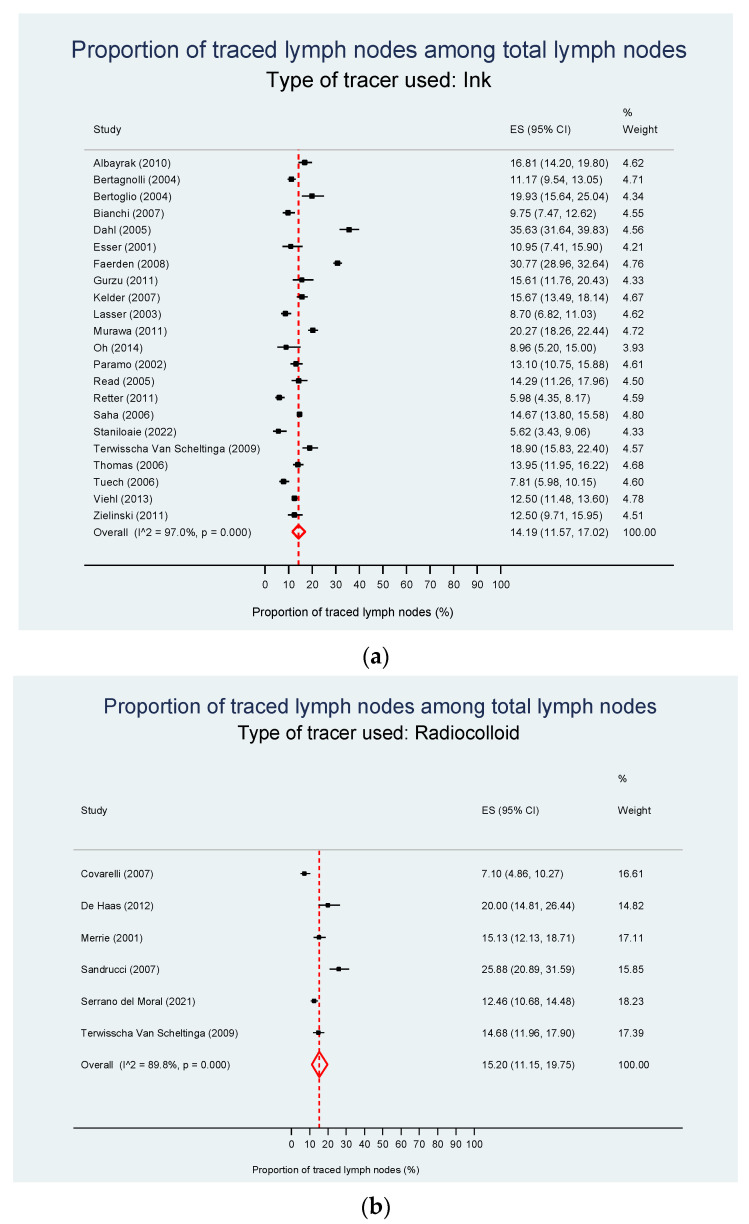

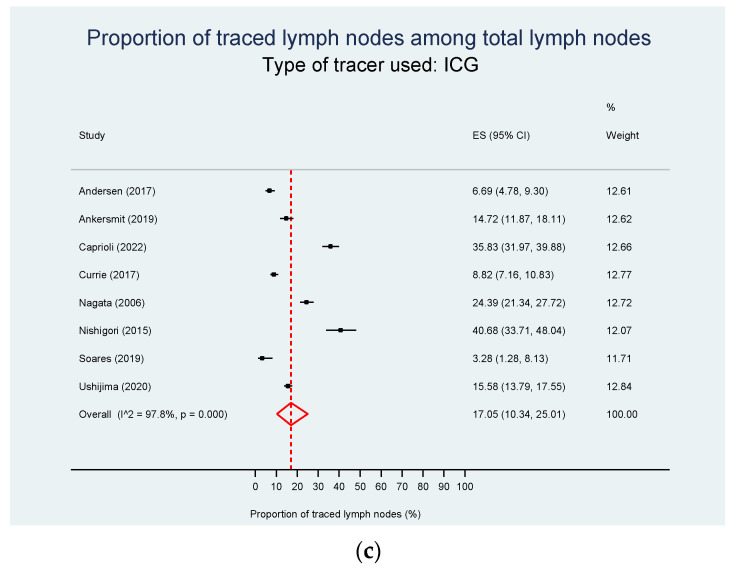
Proportion of traced LNs among total LNs according to tracing agent. (**a**) Ink as tracing agent. The pooled estimate of the proportion of LN nodes is 14.2%. (**b**) Radiocolloid as tracing agent. The pooled estimate of the proportion of traced LNs is 15.2%. (**c**) ICG as tracing agent. The pooled estimate of the proportion of traced LNs is 17.1%. CI—confidence interval; ES—effect size; ICG—indocyanine green [12,16,19,25,26,28,32,35,157,158,159,162,163,165,168,169,170,171,174,175,176,178,180,181,182,183,187,188,189,190,192,193,194,195,197].

From 35 studies, data on the tracers used could be extracted. Twenty-two studies used ink, six radiocolloids, and eight ICG. Lim [179] had to be excluded based on the summarized reporting of different mapping techniques. One study [19] reported results in two groups. The pooled estimate of the traced LNs proportion was stable regardless of the type of tracer: 14.2% in studies using ink, 15.2% in studies using radiocolloid, and 17.1% in studies using ICG with high heterogeneities in all analyses (*p* = 0.740; Figure 3a–c), and Table S3.

**Figure 4:**
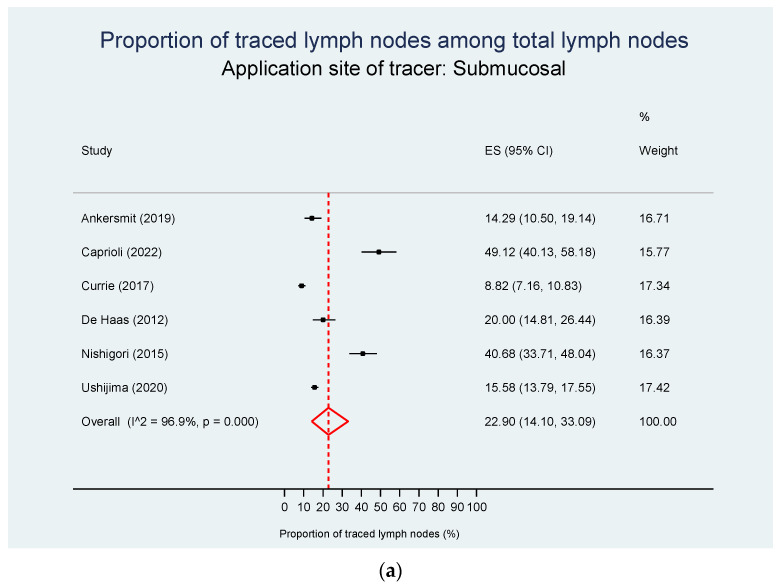

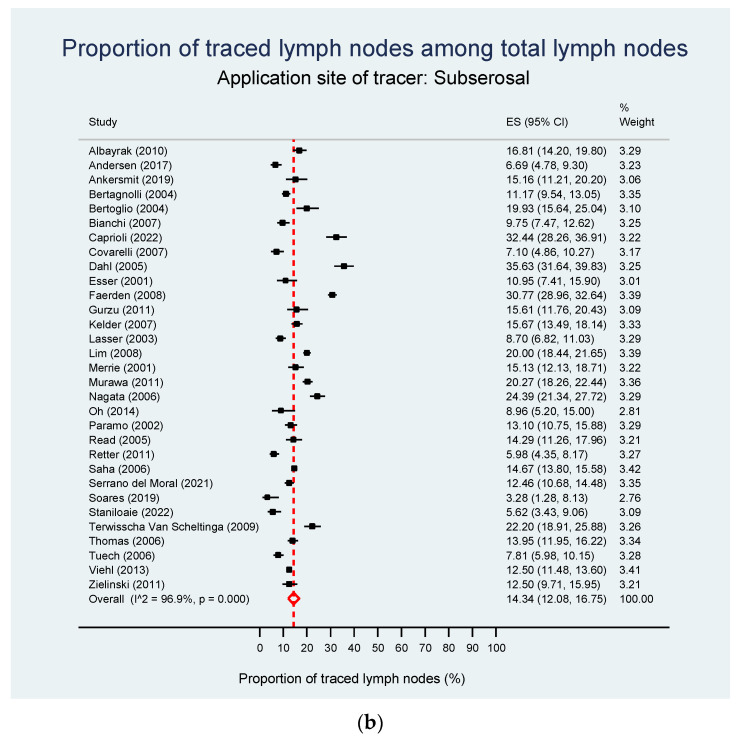
Proportion of tracer-positive LNs among total LNs according to application site of tracer injection. (**a**) Submucosal tracer injection. The pooled estimate of the proportion of traced LNs is 22.9%. (**b**) Subserosal tracer injection. The pooled estimate of the proportion of traced LNs is 14.3%. CI—confidence interval; ES—effect size; ICG—indocyanine green [12,16,19,25,26,28,32,35,157,158,159,162,163,165,168,169,170,171,174,175,176,178,179,180,181,182,183,187,188,190,192,193,194,195,197].

Data on the tracer application site were retrievable from 35 studies, while two cohorts [12,28] were distributed to both groups. Studies not specifying the application site were excluded from this analysis. The pooled estimate of the traced LNs proportion was 22.9% in studies injecting the tracer submucosal and 14.3% for subserosal injection, with high levels of heterogeneity in all analyses (*p* = 0.070, Figure 4a,b) and Table S4.

### 3.3. Abberrant Lymphatic Drainage Detection

All included studies were searched for the mention of aberrant drainage pathways. Of the 58 studies originally meeting the inclusion criteria, 42 mentioned searching for aberrant drainage patterns. Of those 42 studies, 24 found aberrant drainage. For the quantitative analysis, further studies were disqualified due to missing quantification of aberrant drainage [35,161,172,194]. Results are displayed in Table 3.

The reported proportions of successfully mapped patients with aberrant lymphatic drainage ranged from 2.2 [183] to 48.7% [31]. Overall, the pooled estimate of aberrant drainage was 5.1% (2.3, 8.6).

Timing of tracer injection, tracer used, and application sites significantly impacted aberrant drainage detection (*p* < 0.001). Notably, preoperative tracer injection resulted in a pooled rate of 26.3% (11.5, 44.0) compared to 2.5% (0.8, 4.7) following intraoperative tracer injection when all studies searching for aberrant drainage were analyzed. Moreover, ICG was superior in aberrant drainage detection compared to radiocolloids and ink (18.1% (9.2, 28.7) vs. 0.0% (0.0, 1.2) vs. 2.5% (0.9, 4.7)). Submucosal tracer application also resulted in a significantly higher aberrant drainage detection rate compared to subserosal tracer injection (18.5% (3.6, 39.7) vs. 2.0% (0.5, 4.0)).

### 3.4. Individual Patient Data

All individual patient data (IPD) available from the included studies were further analyzed according to factors influencing the effectiveness of LN mapping and aberrant LN detection. Data on tumor characteristics, the timing, and the tracer application site from ten studies could be extracted and included in this analysis [12,16,17,28,29,31,32,159,174,190]. Studies neither searching nor resecting aberrant LNs or not reporting the mapped LN yield did not contribute to the respective analysis.

IPD sets of 210 patients could be extracted from ten studies. One hundred and sixteen patients had right-sided CC, including the caecum, ascending colon, hepatic flexure, and proximal transverse colon. At the same time, 74 suffered from CC located at the splenic flexure, the descending colon, or the sigmoid (Table 4). The majority of tumors were staged pT3 (41.0%), pN0 (51.0%) and had no distant metastasis (31.4%), with a substantial number of 142 cases not reporting the M-stage. Most patients (52.9%) received an intraoperative tracer injection, while the tracer was applicated preoperatively in 82 patients (39.0%). In 17 patients (8.1%), the injection timing remains unclear. The included studies used ink as a tracer for 44 patients (21%) and ICG for 166 patients (79%). No IPD were found for the radiocolloid tracer. The tracer was injected evenly in 96 patients (45.7%) subserosally and in 97 patients (46.2%) submucosally, while data were missing for 17 patients (8.1%). The application site and timing of tracer injection were highly correlated within the IPD. All preoperative applications were performed submucosally, and the vast majority of intraoperative markings were performed subserosally. In only one study’s subcohort, the tracer was applied intraoperatively submucosally in 15 patients [12]. Therefore, the application site of the tracer application was not further analyzed in the IPD.

Data on the traced LN harvest were available from 6 studies involving 112 patients. To avoid interference between parameters, an adjusted linear model was used, adjusting for injection timing, tracer, tumor location, and stage. Preoperative tracer injection resulted in a mean yield of 7.5 ± 6.3 traced LNs, which was significantly higher compared to intraoperative tracer injection (2.8 ± 3.1, regression coefficient −4.488 [95% CI −6.634–−2.543], *p* < 0.001; Table 5). Ink was used in 15 patients, resulting in a mean yield of 1.0 ± 0.8 traced LNs, while ICG traced a mean number of 3.9 ± 4.2 LNs (regression coefficient −1.699 [95% CI −3.751–0.353], *p* = 0.104). The tumor locations also revealed no impact on traced LN yield (regression coefficient 0.797 [95% CI −0.606–2.20], *p* = 0.104). In contrast, earlier tumor stages allowed significantly more effective LN tracing than more advanced T-stages (pTis, pT1, pT2 4.6 ± 4.3 vs. pT3, pT4 2.5 ± 3.6 LN, regression coefficient 1.661 [95% CI 0.272–3.049], *p* = 0.020).

Seven studies searched for aberrant drainage and reported injection timing, tracer, tumor location, and T-stage, involving 121 patients (Table 6). A generalized linear model for adjusted outcome analysis was used, adjusting for the independent parameter. While the tracing methods significantly impacted aberrant lymphatic drainage detection, neither tumor burden nor tumor location were associated with finding an aberrant drainage pattern. Intraoperative tracer application was used in 64 patients and had highly significantly lower odds of finding aberrant drainage compared to preoperative tracer injection, which was present in 57 patients (intraoperative vs. preoperative OR 0.050 [95% CI 0.010–0.176], *p* < 0.001). Furthermore, ink, applied in 44 patients, revealed significantly lower odds of aberrant drainage detection than ICG, and was used in 77 patients (ink vs. ICG OR 0.127 [95% CI 0.018–0.528], *p* = 0.012).

## 4. Discussion

Skip metastases [13,14] and aberrant lymphatic drainage patterns, hence lymph flow inconsistent with the sustentative blood vessels [7,15,16,17], are frequently found in CC. Malignancies can induce not only neoangiogenesis but also lymphangiogenesis, thus surrounding a tumor, increased and newly formed lymphatic flow can exist [20], which differs from the original anatomy. Lymphatic mapping has the potential to unveil those drainage patterns and thereby improve surgical resection when carried out effectively.

This meta-analysis demonstrates that a preoperative tracer application and earlier tumor stages allow a higher mapped LN yield. In contrast, the tracer or application sites have no relevant impact on this ratio. However, for the detection of aberrant LNs, preoperative tracer application, usage of ICG, and a submucosal application demonstrated significantly better results.

Effective LN mapping provides an accurate picture of the tumor’s lymphatic drainage, particularly of those LNs further away and connected via slow-draining lymphatic vessels, as usual following lymphangiogenesis [20]. The tracer application timing was revealed as the strongest predictor of this effectiveness. Preoperative tracer application resulted in a significantly increased rate of traceable LNs with a pooled rate of 30.1%, compared to intraoperative tracer application resulting in 14.1% traceable LNs. Moreover, the traced LN yield was significantly higher when marked preoperatively (*p* = < 0.001), which is in line with results of previous studies [12,119,121].

Aberrant drainage pathways are reported to occur in 2.2–48.7% of patients [15,18,31,37,121,137,183,190,194,198]. However, inappropriate tracing methods have been shown to deteriorate LN mapping results [160] and might fail to identify such patterns. The tracer injection timing has a major impact on aberrant drainage detection, demonstrated in this meta-analysis (intraoperative vs. preoperative: pooled rates 2.5% (0.8, 4.7) vs. 26.3% (11.5, 44.0), *p* < 0.001; IPD: OR 0.050 [95% CI 0.010–0.176], *p* < 0.001). Allowing the tracer more time to travel through the lymphatic system, reaching more distant or slower-connected LNs before detection, enables more effective LN mapping.

A broad range of tracers has been described in the literature. To analyze their effectiveness, we grouped tracers according to detection properties, such as staining tracers (ink, methylene blue, carbon particles, and patient blue), radiocolloid as a radioactive tracer, and ICG as a fluorescent tracer, since the detection methods of the respective tracer group decisively influence their tracing performance. No significant difference was observed in the rate, or the yield of LNs traced (pooled estimate of the proportion of traced LN: ink vs. radiocolloid vs. ICG: 14.2% vs. 15.2% vs. 17.1%; IPD traced LNs: ink vs. ICG, 1.0 ± 0.8 vs. 3.9 ± 4.2, *p* = 0.104), suggesting that all tracers have comparable abilities to travel through the lymphatic system. However, aberrant drainage was significantly more frequently detected using ICG (ink vs. radiocolloid vs. ICG: 2.5% (0.9, 4.7) vs. 0.0% (0.0, 1.2) vs. 18.1% (9.2, 28.7)), which could be confirmed in the IPD analysis (ink vs. ICG OR 0.127 [95% CI 0.018–0.528], *p* = 0.012). Aberrant LNs can easily be missed intraoperatively due to their embeddedness in the mesentery. ICG provides bright visibility even through fatty mesentery, which might enable more precise detection of LNs outside standard resection lines in the surgical site [199]. Further advantages of ICG as the used tracer are the minimal adverse effects reported and that patient and surgeon are not exposed to radioactivity.

The timing of the tracer application is methodically associated with the injection site. Intraoperative application, most commonly subserosal and preoperative tracer application, is performed before skin incision via colonoscopy, submucosally. Therefore, data for a structured investigation of the influence of tracer application sites are rare. This meta-analysis revealed no impact of the injection site on the effectiveness of LN tracing (pooled rate of traced LN: subserosal vs. submucosal 14.3% vs. 22.9%). Only two studies [12,169] used on-table colonoscopy for submucosal tracer application intraoperatively, while all other studies contributing to the submucosal group also performed preoperative tracer injection. Both studies revealed substandard traced lymph node rates, though Ankersmit et al. could prove a higher sensitivity for sentinel lymph node detection after submucosal injection in a comparative study design. Intestinal lymphatic drainage works as dual independent networks in the muscular and the mucosal layer of the intestine wall, which drain to a shared network of collecting ducts. While adenocarcinomas form in the mucosal layer, draining LNs of the mucosal layer and, thus, the tumor may be missed when the tracer is applied subserosally [200,201]. Significantly higher rates of aberrant drainage could be detected when a tracer is injected submucosally. However, this has to be interpreted cautiously due to the increased risk of interference with injection timing.

While the tumor site did not impact the traced LN yield, the tumor stage proved to have a significant association with the amount of traced LNs favoring earlier tumor stages (*p* = 0.020). LN metastases can occlude lymphatic pathways in advanced settings [20], which may lead to fewer traceable LNs. Consequently, LN metastases distant from others might not be traceable by LN mapping. On the other hand, neither tumor location nor tumor stage did influence aberrant lymphatic drainage detection. Lymphangiogenesis as a primary driver of aberrant lymphatic pathways is involved in early tumor stages and takes place in advanced settings as rerouting after lymphatic occlusion [20]. Previous studies postulated the relevance of lymphatic mapping, particular in earlier tumor stages, to correctly stage the disease and reduce recurrence rates. Ultrastaging of traced LNs was proposed to address this topic [73,129,160,163,181] with varying results.

Our data prove the feasibility of tracing a patient’s individual lymphatic drainage, enabling an accurate picture of the lymphatic draining pathway, especially in these relevant earlier stages. This might allow for a tailored multimodal therapeutic approach by refined staging and reducing tumor recurrence. In contrast, neither tumor stage nor site influenced aberrant drainage detection, emphasizing the importance of intraoperative screening for aberrant LNs, which could affect all colon carcinomas equally.

Several aspects limit these findings: significant heterogeneity was present in all traced LN proportion analyses, so these results must be interpreted cautiously. While some studies excluded their learning curves, others did not comment on this or include all patients in which a mapping procedure was carried out. Moreover, the investigation of traced LN rates might be biased since a minimum of 12 pathologically assessed LNs is sufficient according to the ESMO guidelines [202], and it depends on the diligence of the pathologist as to how many LNs are examined beyond that. Unfortunately, data on the measures of dispersion of the absolute number of traced LNs were rare in published literature, so this probably more precise analysis could not be performed. Different standards of lymphatic resection throughout the studies are present, given that standardized CME was only introduced in 2009, and the ongoing debate concerning D2 versus D3 lymphadenectomy. However, this meta-analysis reflects all currently available evidence and approaches the role and methodology of tracing lymphatic drainage scientifically accurately.

## 5. Conclusions

LN mapping has the potential to improve tumor staging and reduce local recurrence by aberrant drainage detection when carried out systematically. Preoperative mapping by colonoscopy and usage of ICG provides the best capacity for accurate visualization of lymphatic drainage. To further investigate the influence of lymphatic mapping on the quality of oncological resections, prospective studies with large patient numbers should be conducted and a standardized protocol adopted for lymphatic mapping prior to surgery to assess whether recurrence rates can be lowered, and long-term survival can be increased.

## Figures and Tables

**Table 1:** Study characteristics.

Study	Study Design	Study Period	Patients	Successful Procedures	Tracer	Injection Timing	Injection Site	T-Stage	Aberrant Drainage
Albayrak [158]	SC	03/04–06/09	38	36	ink	IO	SS	NR	no
Alhassan [36]	SC	NR	15	14	ICG	IO	SS	NR	no
Andersen [159]	MC	02/15–01/16	29	19	ICG	IO	SS	T1–T4	yes
Ankersmit [12]	SC	NR	29	26	ICG	IO	SM & SS	T1–T4	–
Bellido Luque [37]	SC	NR	16	16	ICG	IO	NR	NR	yes
Bembenek [160]	MC	01/03–08/05	315	268	ink	IO	SS	NR	yes
Bendavid [161]	SC	09/99–05/00	20	18	ink	IO	SS	NR	yes
Bertagnolli [162]	MC	02/99–01/03	72	66	ink	IO	SS	T1–T4	–
Bertoglio [163]	SC	02/99–01/03	20	19	ink	IO	SS	NR	–
Bianchi [164]	SC	06–NR	61	57	ink	IO	SS	NR	yes
Bianchi [165]	SC	03/04–12/05	22	22	ink	IO	SS	T1–T4	yes
Caprioli [28]	SC	04/17–08/18	32	32	ICG	IO & PO	SM & SS	T1–T4	yes
Carrara [166]	SC	04/14–NR	95	92	ICG	IO	SS	T1–T4	–
Chan [167]	SC	08/05–07/06	19	18	ink	IO	SS	NR	–
Chand [18]	SC	03–06/17	10	10	ICG	IO	SS	T1, T3, T4	yes
Covarelli [168]	SC	NR	20	19	RC	IO	SS	NR	no
Currie [169]	SC	08/13–08/15	30	27	ICG	IO	SM	T1–T4	no
Dahl [35]	SC	NR	30	30	ink	IO	SS	NR	yes
Esser [170]	SC	01/98–11/99	26	14	ink	IO	SS	NR	–
Faerden [171]	SC	12/00–09/05	200	185	ink	IO	SS	T1–T4	–
Feng [17]	SC	NR	27	27	ICG & ink	PO	SM	T1–T4	yes
Goo [172]	SC	NR	156	156	ICG	PO	SM	T1–T4	no
Gundogdu [173]	SC	10/10–10/11	84	74	ink	IO	SS	T2–T4	no
Gurzu [174]	SC	Dec. 2009–Dec. 2010	15	14	ink	IO	SS	T2, T3	no
De Haas [175]	SC	NR	14	12	RC	PO	SM	T2, T3	no
Ho [29]	SC	NR	21	18	ICG	PO	SM	T1–T4	yes
Kakizoe [156]	SC	07/13–12/18	72	71	ICG	IO	NR	T1–T4	–
Kinoshita [30]	SC	10/18–03/21	56	43	ICG	IO	SS	T1–T4	yes
Kelder [176]	MC	05/02–05/05	69	67	ink	IO	SS	T1–T4	yes
Kolev [177]	SC	NR	48	48	ink	IO	NR	NR	yes
Lasser [178]	SC	02/01–08/02	30	30	ink	IO	SS	Tis–T4	yes
Lim [179]	SC	09/98–04/06	120	119	ink & RC	IO	SS	T1–T4	no
Merrie [180]	SC	NR	26	23	RC	IO	SS	NR	–
Murawa [181]	SC	05/05–09/10	100	99	ink	IO	SS	Tis–T3	yes
Nagata [25]	SC	07/02–12/04	37	33	ICG	IO	SS	T1–T3	–
Nishigori [16]	SC	03/13–06/14	11	11	ICG	PO	SM	Tis–T4	yes
Oh [182]	SC	02/11–10/12	11	10	ink	IO	SS	T1, T3, T4	–
Paramo [183]	SC	06/99–08/01	55	45	ink	IO	SS	T1–T3	yes
S. Park [184]	SC	06/16–12/17	25	25	ICG	PO	SM	NR	yes
J.S. Park [185]	SC	05/06–06/08	37	30	ink	IO	SS	NR	–
Patten [186]	SC	02/00-NR	57	56	ink & RC	IO	SS	T1–T4	no
Petz [15]	SC	07/16–07/20	50	50	ICG	PO	SM	NR	yes
Read [187]	SC	NR	38	30	ink	IO	SS	NR	no
Ribero [31]	SC	04/19–05/21	70	70	ICG	PO	SM	T1–T4	yes
Retter [188]	SC	08/05–01/08	31	28	ink	IO	NR	T1–T4	no
Saha [26]	MC	1996–2004	408	405	ink	IO	SS	T1–T4	–
Sandrucci [189]	SC	02/01–12/04	30	30	RC	IO & PO	SM & SS	Tis–T2	no
Serrano del Moral [157]	SC	10/10–03/14	72	62	RC	IO	NR	T1–T4	no
Soares [190]	SC	10–12/18	5	4	ICG	IO	SS	T1–T4	no
Staniloaie [32]	SC	01/18–02/20	26	18	Ink	IO	SS	T1–T4	no
Tang [191]	SC	03/16–12/16	40	40	ink	IO	SS	NR	–
Terwisscha Van Scheltinga [19]	SC	08/03–07/04	53	49	ink & RC	IO	SS	NR	–
Thomas [192]	SC	03/99–07/05	69	64	ink	IO	SS	NR	no
Tuech [193]	SC	NR	34	33	ink	IO	SS	T1–T3	yes
Ushijima [194]	SC	10/16–07/17	57	43	ICG	PO	SM	NR	yes
Viehl [195]	MC	NR	174	155	ink	IO	SS	T1–T4	–
Vîlcea [196]	SC	NR	22	19	ink	IO	SS	NR	–
Zielinski [197]	SC	NR	42	36	ink	IO	SS	T1–T4	no

SC—single center study; MC—multicenter study; NR—not reported; ink—ink (methylene blue and patient blue); ICG—indocyanine green; RC—radiocolloid; IO—intraoperative tracer injection; PO—preoperative tracer injection; SS—subserosal application; SM—submucosal application; –—not searched for aberrant drainage; no—found no aberrant drainage while searching for it; yes—reports finding aberrant drainage.

**Table 2:** Quality assessment using the QUADAS-2 tool.

Study	Risk of Bias				Applicability Concerns		
	Patient Selection ^(a)^	Index Test ^(b)^	Reference Standard ^(c)^	Flow and Timing ^(d)^	Patient Selection ^(e)^	Index Test ^(f)^	Reference Standard ^(g)^
Albayrak [158]	?	☺	☺	☺	☺	☺	☺
Alhassan [36]	?	☺	?	☺	☺	☺	☺
Andersen [159]	☺	☺	☺	☺	☺	☺	☺
Ankersmit [12]	☺	☺	☹	☺	☺	☺	☺
Bellido Luque [37]	?	☺	☺	☺	☺	☺	☺
Bembenek [160]	?	☺	☹	☺	☺	☺	☺
Bendavid [161]	?	☺	☹	☺	☺	☺	☺
Bertagnolli [162]	?	☺	☹	☺	☺	☺	☺
Bertoglio [163]	?	☺	☹	☺	☺	☺	☺
Bianchi [164]	?	☺	☺	☺	☺	☺	☺
Bianchi [165]	?	☺	☺	☺	☺	☺	☺
Caprioli [28]	?	☺	☺	☺	☹	☺	☺
Carrara [166]	☺	☺	☹	☺	☹	☺	☺
Chan [167]	☺	☺	☹	☺	☺	☺	☺
Chand [18]	☺	☺	?	☺	☺	☺	☺
Covarelli [168]	?	☺	☹	☺	☺	☺	☺
Currie [169]	☺	☺	☹	☺	☺	☺	☺
Dahl [35]	?	☺	☺	☺	☺	☺	☺
Esser [170]	☺	☺	☺	☺	☺	☺	☺
Faerden [171]	☺	☺	☹	☺	☹	☺	☺
Feng [17]	☺	☺	☺	☺	☺	☺	☺
Goo [172]	?	☺	?	☺	☺	☺	☺
Gundogdu [173]	☺	☺	?	☺	☺	☺	☺
Gurzu [174]	?	☺	☹	☺	☺	☺	☺
De Haas [175]	☺	☺	☹	☺	☺	☺	☺
Ho [29]	?	☺	☺	☺	☺	☺	☺
Kakizoe [156]	☺	☺	☺	☺	☺	☺	☺
Kinoshita [30]	?	☺	☹	☺	☺	☺	☺
Kelder [176]	?	☺	☹	☺	☺	☺	☺
Kolev [177]	☺	☺	☹	☺	☺	☺	☺
Lasser [178]	☺	☺	☹	☺	☹	☺	☺
Lim [179]	☺	☺	☹	☺	☺	☺	☺
Merrie [180]	?	☺	☹	☺	☺	☺	☺
Murawa [181]	☺	☺	☹	☺	☺	☺	☺
Nagata [25]	?	☺	☺	☺	☹	☺	☺
Nishigori [16]	?	☺	☺	☺	☺	☺	☺
Oh [182]	?	☺	☺	☺	☺	☺	☺
Paramo [183]	☺	☺	☹	☺	☺	☺	☺
S. Park [184]	?	☺	☺	☺	☺	☺	☺
J.S. Park [185]	☺	☺	☹	☺	☺	☺	☺
Patten [186]	☺	☺	☺	☺	☺	☺	☺
Petz [15]	☺	☺	☺	☺	☺	☺	☺
Read [187]	?	☺	☺	☺	☺	☺	☺
Ribero [31]	?	☺	☺	☺	☹	☺	☺
Retter [188]	?	☺	☹	☺	☺	☺	☺
Saha [26]	☺	☺	☹	☺	☺	☺	☺
Sandrucci [189]	☺	☺	☺	☺	☺	☺	☺
Serrano del Moral [157]	?	☺	☹	☺	☺	☺	☺
Soares [190]	☺	☺	☺	☺	☺	☺	☺
Staniloane [32]	?	☺	☺	☺	☹	☺	☺
Tang [191]	☺	☺	☺	☺	☺	☺	☺
Terwisscha Van Scheltinga [19]	?	☺	☹	☺	☺	☺	☺
Thomas [192]	?	☺	☹	☺	☺	☺	☺
Tuech [193]	☺	☺	☹	☺	☹	☺	☺
Ushijima [194]	☺	☺	☺	☺	☺	☺	☺
Viehl [195]	☺	☺	☹	☺	☺	☺	☺
Vîlcea [196]	☺	☺	☺	☺	☺	☺	☺
Zielinski [197]	?	☺	☹	☺	☺	☺	☺

☺ = low risk of bias/low applicability concerns, ☹ = high risk of bias/applicability concerns, ? = unclear risk of bias/applicability concerns. (a) Patient selection according to QUADAS-2. (b) Review specific index test: performance of oncological surgery without knowing LN status. (c) Review specific applicability assessment: number of metastatic LNs without additional staging. (d) Flow and timing according to QUADAS-2. (e) Review specific applicability of patient selection: Location of tumor; ☹ includes rectosigmoid carcinoma. (f) Review specific index test: tracer application peri-tumorous in vivo followed by standard pathological LN assessment. (g) Review specific reference standard: Could the conduct of additional staging tests have introduced bias?

**Table 3:** Detection rate of aberrant lymphatic drainage by lymph node mapping.

	Number of Studies	Pooled Rate Derived from Meta-Analysis (%)	*p*-Value
Overall	38	5.1 (2.3, 8.6)	-
Timing of tracer injection			
Intraoperative	30	2.5 (0.8, 4.7)	**<0.001**
Preoperative	5	26.3 (11.5, 44.0)	
Tracer			
Ink	20	2.5 (0.9, 4.7)	**<0.001**
Radiocolloid	4	0.0 (0.0, 1.2)	
ICG	13	18.1 (9.2, 28.7)	
Application site of tracer injection			
Submucosal	6	18.5 (3.6, 39.7)	**<0.001**
Subserosal	27	2.0 (0.5, 4.0)	

Numbers are indicated as pooled rate of patients and 95% CI. *p*-values in bold indicate statistical significance between cohorts. CI—confidence interval; ICG—indocyanine green.

**Table 4:** Overview of individual patient data.

	n	%
Study		
Ankersmit [12]	29	13.8
Andersen [159]	29	13.8
Feng [17]	27	12.9
Gurzu [174]	15	7.1
Nishigori [16]	11	5.2
Soares [190]	5	2.4
Staniloaie [32]	15	7.1
Ribero [31]	39	18.6
Ho [29]	17	8.1
Caprioli [28]	23	11.0
Tumor location		
Right-sided	116	55.2
caecum	31	14.8
ascending colon	70	33.3
hepatic flexure	1	0.5
transverse colon	14	6.7
Left-sided	74	35.2
splenic flexure	3	1.4
descending colon	10	4.8
sigmoid colon	61	29.0
data missing	20	9.5
T-stage		
Tis	3	1.4
T1	12	5.7
T2	29	13.8
T3	86	41.0
T4	20	9.5
N-stage		
N0	107	51.0
N1	40	19.0
N2	20	9.5
data missing	43	20.5
Lymphnode harvest	24.2 (±14.6)	
M		
M0	66	31.4
M1	2	1.0
data missing	142	67.6
Timing		
intraoperative	111	52.9
preoperative	82	39.0
data missing	17	8.1
Tracer		0.0
ink	44	21.0
ICG	166	79.0
Tracer application site		
submucosal	97	46.2
subserosal	96	45.7
data missing	17	8.1

Numbers are presented as absolute numbers and percentages; LN harvest is given as a mean with standard deviation in parenthesis. ICG—indocyanine green, M0—M2 indicate distant metastases, N0—N2 indicate nodal status, T1—T4 indicate tumor stadium.

**Table 5:** Adjusted analysis for traced lymph nodes in individual patients.

	Number of Patients	Mean	Standard Deviation	Regression Coefficient	95% CI	*p*-Value
Timing						
Intraoperative	96	2.8	3.1	−4.488	−6.634–−2.543	**<0.001**
Preoperative	16	7.5	6.3			
Tracer						
Ink	15	1.0	0.8	−1.699	−3.751–0.353	0.104
ICG	97	3.9	4.2			
Tumor location						
Right-sided	69	3.7	4.4	0.797	−0.606–2.200	0.263
Left-sided	43	3.1	3.5			
T-stage						
pTis—pT1—pT2	53	4.6	4.3	1.661	0.272–3.049	**0.020**
pT3–pT4	59	2.5	3.6			

*p*-values in bold indicate statistical significance between cohorts. CI—confidence interval of regression coefficient, ICG—indocyanine green, t—tumor stage.

**Table 6:** Adjusted analysis of aberrant drainage derived from individual patient data.

Parameter	OR [95% CI]	Regression Coefficient	*p*-Value
Timing			
Intraoperative vs. Preoperative	0.050 [0.010–0.176]	−2.989	**<0.001**
Tracer			
Ink vs. ICG	0.127 [0.018–0.528]	−2.064	**0.012**
Tumor localization			
Right-sided vs. Left-sided	0.449 [0.118–1.533]	−0.801	0.212
T-stage			
pTis, pT1, pT2 vs. pT3, T4	1.163 [0.333–3.919]	0.151	0.808

*p*-values in bold indicate statistical significance between cohorts. CI—confidence interval, OR—odds ratio.

## Data Availability

The data presented in this study are available on request from the corresponding author.

## Supplementary Materials

The following supporting information can be downloaded at: https://www.mdpi.com/article/10.3390/cancers15123196/s1, Data S1: Search strategy. Table S1: Quality assessment according to QUADAS-2 with partly review-specific tailored questions. Table S2: Traced LNs of all LNs according to injection timing. Table S3: Traced LNs of all LNs according to tracer. Table S4: Traced LNs of all LNs according to application site of tracer injection.
