# Supplementary material for: Lymphatic Mapping in Colon Cancer Depending on Injection Time and Tracing Agent: A Systematic Review and Meta-Analysis of Prospective Designed Studies
Source: Cancers (Basel). 2023 Jun 15;15(12):3196. doi: 10.3390/cancers15123196 (PMC10296374; doi:10.3390/cancers15123196)
Supplement: Supplementary file 1 [file cancers-15-03196-s001.zip › cancers-2434681-supplementary.pdf]

Supplementary data
Lymphatic mapping in colon cancer depending on injection time and tracing agent: a systematic review and meta-analysis of prospective designed studies

Data S1: Search strategy

Search strategy: Searched databases include Medline, Web of Science, including "forward cited search" [1-3] and Embase (through OVID), register searched include Cochrane and PROSPERO. Originally, a Pubmed search string was validated by preliminarily finding already known studies and translated via SR-accelerators polyglot search [4] to match other databases. All searches were conducted on July 20th, 2021, the original search string can be found in the supplementary data. The process of this search is displayed in table 1. A second search using the same search strategies was conducted on February 13th, 2023, with the objective of finding newly published studies. Five newly published studies [5-9] were included. Reviews [10-16] concerning similar research topics were manually searched for possible missed publications. One study [17] was found through this process.

The full Pubmed search string with the restriction to "human" was:
(("colon carcinoma"[Text Word] OR "colon cancer"[Text Word] OR "colon neoplasms"[Text Word] OR "Ileal Neoplasms"[Text Word] OR "neuroendocrine tumor"[Text Word] OR "neuroendocrine tumour"[Text Word] OR "Colorectal Neoplasms"[MeSH Terms] OR "Neuroendocrine Tumors"[MeSH Terms:noexp] OR "Ileal Neoplasms"[MeSH Terms]) AND ("lymphatic visualiza*"[Text Word] OR "lymphatic mapp*"[Text Word] OR "Lymphatic System"[MeSH Terms] OR "Lymph Nodes"[MeSH Terms] OR "Sentinel Lymph Node Biopsy"[MeSH Terms] OR "Lymph Nodes"[MeSH Terms] OR "Sentinel Lymph Node"[MeSH Terms] OR "lymphatic disease"[Text Word] OR "Lymphatic Metastasis"[Text Word] OR "lymph node metastasis"[Text Word] OR "Lymphatic Metastasis"[MeSH Terms] OR "Sentinel Lymph Node"[MeSH Terms]) AND ("fluorescence marking"[Text Word] OR "ICG marking"[Text Word] OR "ICG"[Text Word] OR "near-infrared fluorescence"[Text Word] OR "imaging-guided"[Text Word] OR "Fluorescence"[MeSH Terms] OR "Optical Imaging"[MeSH Terms] OR "Indocyanine Green"[MeSH Terms])) OR (("colon carcinoma"[Text Word] OR "colon cancer"[Text Word] OR "colon neoplasms"[Text Word] OR "Ileal Neoplasms"[Text Word] OR "neuroendocrine tumor"[Text Word] OR "neuroendocrine tumour"[Text Word] OR "Colorectal Neoplasms"[MeSH Terms] OR "Neuroendocrine Tumors"[MeSH Terms:noexp] OR "Ileal Neoplasms"[MeSH Terms]) AND ("lymphatic visualiza*"[Text Word] OR "lymphatic mapp*"[Text Word] OR "Lymphatic System"[MeSH Terms] OR "Lymph Nodes"[MeSH Terms] OR "Sentinel Lymph Node Biopsy"[MeSH Terms] OR "Lymph Nodes"[MeSH Terms] OR "Sentinel Lymph Node"[MeSH Terms] OR "lymphatic disease"[Text Word] OR "Lymphatic Metastasis"[Text Word] OR "lymph node metastasis"[Text Word] OR "Lymphatic Metastasis"[MeSH Terms] OR "Sentinel Lymph Node"[MeSH Terms]) AND ((("ink marking"[Text Word] OR "ink tattooing"[Text Word] OR "tattooing"[Text Word] OR "dye"[Text Word] OR "isosulfan blue"[Text Word] OR "methylene

blue"[Text Word] OR "blue dye"[Text Word]) AND "patent blue"[Text Word]) OR "blue"[Text Word] OR "Optical Imaging"[MeSH Terms] OR "Ink"[MeSH Terms] OR "iso-sulfan blue"[Supplementary Concept])) OR (("colon carcinoma"[Text Word] OR "colon cancer"[Text Word] OR "colon neoplasms"[Text Word] OR "Ileal Neoplasms"[Text Word] OR "neuroendocrine tumor"[Text Word] OR "neuroendocrine tumour"[Text Word] OR "Colorectal Neoplasms"[MeSH Terms] OR "Neuroendocrine Tumors"[MeSH Terms:noexp] OR "Ileal Neoplasms"[MeSH Terms]) AND ("lymphatic visualiza*"[Text Word] OR "lymphatic mapp*"[Text Word] OR "Lymphatic System"[MeSH Terms] OR "Lymph Nodes"[MeSH Terms] OR "Sentinel Lymph Node Biopsy"[MeSH Terms] OR "Lymph Nodes"[MeSH Terms] OR "Sentinel Lymph Node"[MeSH Terms] OR "lymphatic disease"[Text Word] OR "Lymphatic Metastasis"[Text Word] OR "lymph node metastasis"[Text Word] OR "Lymphatic Metastasis"[MeSH Terms] OR "Sentinel Lymph Node"[MeSH Terms]) AND ("radiocolloid"[Text Word] OR "technetium-99m"[Text Word] OR "Tc-99m"[Text Word] OR "99m"[All Fields] OR "radioisotope"[Text Word] OR "radiotracer"[Text Word] OR "lymphatic tracer"[Text Word] OR "tracer"[Text Word] OR "superparamagnetic"[Text Word] OR "iron oxide"[Text Word] OR "magnetic technique"[Text Word] OR "Technetium"[MeSH Terms]))

The search string for Embase through OVID and Web of Science was translated by polyglot-translator, the search string for Cochrane consists of: (Colon OR colorectal) AND (cancer OR carcinoma) and the search string for PROSPERO of: (Colon OR colorectal) AND (cancer OR carcinoma) AND (lymphatic OR mapping OR sentinel).

Table S1: Quality assessment according to QUADAS-2 with partly review specific tailored questions

	Risk of Bias				Applicability concerns		
	a) Patient selection according to QUADAS-2	b) Review specific index test	c) Review specific applicability assessment	d) Flow and timing according to QUADAS-2	e) Review specific applicability of patient selection	f) Review specific index test	g) Review specific reference standard
	Was a consecutive sample of patients enrolled?<br><br>Did the study avoid inappropriate exclusions?	Was an oncologic surgery performed?	Was the number of metastatic LNs without additional staging given?	Was there an appropriate[3] interval between index test[1] and reference standard[2]?<br><br>Did all patients receive a reference standard[2]?<br><br>Did all patients receive the same reference standard[2]?	Were rectum or rectosigmoid tumours included?	Was the tracer applicated peri-tumourous in vivo followed by pathological LN assessment?	Was LN data extractable without additional staging methods that could have introduced bias through more sensitive proof of metastasis?
☺ = low risk of bias/low applicability concerns	Yes, to all questions	Yes	Yes	Yes, to all questions	No	Yes	yes
☹ = high risk of bias/applicability concerns	No, to either question	No	Number of metastatic lymph nodes without addiotional staging methods retrieveable through text, supplementary data or contact of authors	No, to any question	Yes, rectum and/or rectosigmoid tumours included, but CC data extractable	No	No
? = unclear risk of bias/applicability concerns	No mention of consecutive patient inclusion	Not mentioned	No mention of pathologic staging method – presumably standard assessement	Not mentioned	Not specified	Not specified	Not specified

[1] Index Test: tracer application, [2] Reference Standard: tracer detection; [3] appropriate: between minutes to three days;

Table S2: Table of Analysis according to timing of tracer injection

**Timing of injection**	**DURING SURGERY**		**BEFORE SURGERY**	
**Study**	**Rate in individual studies (95% CI)**	**Weights meta-analysis (%)**	**Rate in individual studies (95% CI)**	**Weights meta-analysis (%)**
Albayrak (2010)	16.8 (14.2, 19.8)	3.2		
Andersen (2017)	6.7 (4.8, 9.3)	3.1		
Ankersmit (2019)	14.7 (11.9, 18.1)	3.1		
Bertagnolli (2004)	11.2 (9.5, 13.1)	3.2		
Bertoglio (2004)	19.9 (15.6, 25.0)	3.0		
Bianchi (2007)	9.8 (7.5, 12.6)	3.1		
Caprioli (2022)	32.4 (28.3, 36.9)	3.1	49.1 (40.1, 58.2)	24.3
Covarelli (2007)	7.1 (4.9, 10.3)	3.1		
Currie (2017)	8.8 (7.2, 10.8)	3.2		
Dahl (2005)	35.6 (31.6, 39.8)	3.1		
De Haas (2012)			20.0 (14.8, 26.4)	24.9
Esser (2001)	11.0 (7.4, 15.9)	2.9		
Faerden (2008)	30.8 (29.0, 32.6)	3.3		
Gurzu (2011)	15.6 (11.8, 20.4)	3.0		
Kelder (2007)	15.7 (13.5, 18.1)	3.2		
Lasser (2003)	8.7 (6.8, 11.0)	3.2		
Lim (2008)	20.0 (18.4, 21.7)	3.3		
Merrie (2001)	15.1 (12.1, 18.7)	3.1		
Murawa (2011)	20.3 (18.3, 22.4)	3.3		
Nagata (2006)	24.4 (21.3, 27.7)	3.2		
Nishigori (2015)			40.7 (33.7, 48)	24.9
Oh (2014)	9.0 (5.2, 15.0)	2.7		
Paramo (2002)	13.1 (10.8, 15.9)	3.2		
Read (2005)	14.3 (11.3, 18.0)	3.1		
Retter (2011)	6.0 (4.4, 8.2)	3.2		
Saha (2006)	14.7 (13.8, 15.6)	3.3		
Serrano del Moral (2021)	12.5 (10.7, 14.5)	3.2		
Soares (2019)	3.3 (1.3, 8.1)	2.7		
Staniloaie (2022)	5.6 (3.4, 9.1)	3.0		
Terwisscha Van Scheltinga (2009)	22.2 (18.9, 25.9)	3.2		
Thomas (2006)	14.0 (12.0, 16.2)	3.2		
Tuech (2006)	7.8 (6.0, 10.2)	3.2		
Ushijima (2020)			15.6 (13.8, 17.6)	25.9
Viehl (2013)	12.5 (11.5, 13.6)	3.3		
Zielinski (2011)	12.5 (9.7, 16.0)	3.1		
**Pooled rate (95% CI)**	**14.1 (11.9, 16.5)**	**100**	**30.1 (15.4, 47.3)**	**100**
**Heterogeneity**	**I^2=96.9%, p<0.001**		**I^2=97.0%, p<0.001**	

Table S3: Table of Analysis according to tracer used

Tracer used	INK		RADIOCOLLOID		ICG	
**Study**	**Rate in individual studies (95% CI)**	**Weights meta-analysis (%)**	**Rate in individual studies (95% CI)**	**Weights meta-analysis (%)**	**Rate in individual studies (95% CI)**	**Weights meta-analysis (%)**
Albayrak (2010)	16.8 (14.2, 19.8)	4.6				
Andersen (2017)					6.7 (4.8, 9.3)	12.6
Ankersmit (2019)					14.7 (11.9, 18.1)	12.6
Bertagnolli (2004)	11.2 (9.5, 13.1)	4.7				
Bertoglio (2004)	19.9 (15.6, 25.0)	4.3				
Bianchi (2007)	9.8 (7.5, 12.6)	4.6				
Caprioli (2022)					35.8 (32.0, 39.9)	12.7
Covarelli (2007)			7.1 (4.9, 10.3)	16.6		
Currie (2017)					8.8 (7.2, 10.8)	12.8
Dahl (2005)	35.6 (31.6, 39.8)	4.6				
De Haas (2012)			20.0 (14.8, 26.4)	14.8		
Esser (2001)	11.0 (7.4, 15.9)	4.2				
Faerden (2008)	30.8 (29.0, 32.6)	4.8				
Gurzu (2011)	15.6 (11.8, 20.4)	4.3				
Kelder (2007)	15.7 (13.5, 18.1)	4.7				
Lasser (2003)	8.7 (6.8, 11.0)	4.6				
Merrie (2001)			15.1 (12.1, 18.7)	17.1		
Murawa (2011)	20.3 (18.3, 22.4)	4.7				
Nagata (2006)					24.4 (21.3, 27.7)	12.7
Nishigori (2015)					40.7 (33.7, 48.0)	12.1
Oh (2014)	9.0 (5.2, 15.0)	3.9				
Paramo (2002)	13.1 (10.8, 15.9)	4.6				
Read (2005)	14.3 (11.3, 18.0)	4.5				
Retter (2011)	6.0 (4.4, 8.2)	4.6				
Saha (2006)	14.7 (13.8, 15.6)	4.8				
Sandrucci (2007)			25.9 (20.9, 31.6)	15.9		
Serrano del Moral (2021)			12.5 (10.7, 14.5)	18.2		
Soares (2019)					3.3 (1.3, 8.1)	11.7
Staniloaie (2022)	5.6 (3.4, 9.1)	4.3				
Terwisscha Van Scheltinga (2009)	18.9 (15.8, 22.4)	4.6	14.7 (12.0, 17.9)	17.4		
Thomas (2006)	14.0 (12.0, 16.2)	4.7				
Tuech (2006)	7.8 (6.0, 10.2)	4.6				
Ushijima (2020)					15.6 (13.8, 17.6)	12.8
Viehl (2013)	12.5 (11.5, 13.6)	4.8				
Zielinski (2011)	12.5 (9.7, 16.0)	4.5				
**Pooled rate (95% CI)**	**14.2 (11.6, 17.0)**	**100**	**15.2 (11.1, 19.7)**	**100**	**17.1 (10.3, 25.0)**	**100**
**Heterogeneity**	**I^2=97.0%, p<0.001**		**I^2=89.8%, p<0.001**		**I^2=97.8%, p<0.001**	

Table S4: Table of Analysis according to location of tracer injection

**Location of injection**	**SUBMUCOSAL**		**SUBSEROSAL**	
**Study**	**Rate in individual studies (95% CI)**	**Weights meta-analysis (%)**	**Rate in individual studies (95% CI)**	**Weights meta-analysis (%)**
Albayrak (2010)			16.8 (14.2, 19.8)	3.3
Andersen (2017)			6.7 (4.8, 9.3)	3.2
Ankersmit (2019)	14.3 (10.5, 19.1)	16.7	15.2 (11.2, 20.2)	3.1
Bertagnolli (2004)			11.2 (9.5, 13.1)	3.4
Bertoglio (2004)			19.9 (15.6, 25.0)	3.1
Bianchi (2007)			9.8 (7.5, 12.6)	3.3
Caprioli (2022)	49.1 (40.1, 58.2)	15.8	32.4 (28.3, 36.9)	3.2
Covarelli (2007)			7.1 (4.9, 10.3)	3.2
Currie (2017)	8.8 (7.2, 10.8)	17.3		
Dahl (2005)			35.6 (31.6, 39.8)	3.3
De Haas (2012)	20.0 (14.8, 26.4)	16.4		
Esser (2001)			11.0 (7.4, 15.9)	3.0
Faerden (2008)			30.8 (29.0, 32.6)	3.4
Gurzu (2011)			15.6 (11.8, 20.4)	3.1
Kelder (2007)			15.7 (13.5, 18.1)	3.3
Lasser (2003)			8.7 (6.8, 11.0)	3.3
Lim (2008)			20.0 (18.4, 21.7)	3.4
Merrie (2001)			15.1 (12.1, 18.7)	3.2
Murawa (2011)			20.3 (18.3, 22.4)	3.4
Nagata (2006)			24.4 (21.3, 27.7)	3.3
Nishigori (2015)	40.7 (33.7, 48)	16.4		
Oh (2014)			9.0 (5.2, 15.0)	2.8
Paramo (2002)			13.1 (10.8, 15.9)	3.3
Read (2005)			14.3 (11.3, 18.0)	3.2
Retter (2011)			6.0 (4.4, 8.2)	3.3
Saha (2006)			14.7 (13.8, 15.6)	3.4
Serrano del Moral (2021)			12.5 (10.7, 14.5)	3.4
Soares (2019)			3.3 (1.3, 8.1)	2.8
Staniloaie (2022)			5.6 (3.4, 9.1)	3.1
Terwisscha Van Scheltinga (2009)			22.2 (18.9, 25.9)	3.3
Thomas (2006)			14.0 (12.0, 16.2)	3.3
Tuech (2006)			7.8 (6.0, 10.2)	3.3
Ushijima (2020)	15.6 (13.8, 17.6)	17.4		
Viehl (2013)			12.5 (11.5, 13.6)	3.4
Zielinski (2011)			12.5 (9.7, 16.0)	3.2
**Pooled rate (95% CI)**	**22.9 (14.1, 33.1)**	**100**	**14.3 (12.1, 16.8)**	**100**
**Heterogeneity**	**I^2=96.9%, p<0.001**		**I^2=96.9%, p<0.001**	

Sources:

1. Nagata, K., et al., *Laparoscopic sentinel node mapping for colorectal cancer using infrared ray laparoscopy.* Anticancer Res, 2006. **26**(3b): p. 2307-11.
2. Saha, S., et al., *A multicenter trial of sentinel lymph node mapping in colorectal cancer: prognostic implications for nodal staging and recurrence.* Am J Surg, 2006. **191**(3): p. 305-10.
3. Nishigori, N., et al., *Visualization of Lymph/Blood Flow in Laparoscopic Colorectal Cancer Surgery by ICG Fluorescence Imaging (Lap-IGFI).* Ann Surg Oncol, 2016. **23 Suppl 2**: p. S266-74.
4. Clark, J.M., et al., *Improving the translation of search strategies using the Polyglot Search Translator: a randomized controlled trial.* J Med Libr Assoc, 2020. **108**(2): p. 195-207.
5. Ho, M.F., et al., *Personalized laparoscopic resection of colon cancer with the use of indocyanine green lymph node mapping: Technical and clinical outcomes.* Asian journal of endoscopic surgery, 2022. **15**(3): p. 563-568.
6. Caprioli, M., et al., *Fluorescence-guided nodal navigation during colectomy for colorectal cancer.* Minim Invasive Ther Allied Technol, 2022. **31**(6): p. 879-886.
7. Staniloaie, D., et al., *Role of methylene blue in detecting the sentinel lymph node in colorectal cancer: In vivo vs. ex vivo technique.* Experimental and Therapeutic Medicine, 2022. **23**(1).
8. Kinoshita, H., et al., *Timing of real-time indocyanine green fluorescence visualization for lymph node dissection during laparoscopic colon cancer surgery.* Langenbecks Arch Surg, 2023. **408**(1): p. 38.
9. Ribero, D., et al., *ICG-Guided Lymphadenectomy during Surgery for Colon and Rectal Cancer-Interim Analysis of the GREENLIGHT Trial.* Biomedicines, 2022. **10**(3).
10. Ankersmit, M., et al., *Near-infrared fluorescence imaging for sentinel lymph node identification in colon cancer: a prospective single-center study and systematic review with meta-analysis.* Tech Coloproctol, 2019. **23**(12): p. 1113-1126.
11. Burghgraef, T.A., et al., *In vivo sentinel lymph node identification using fluorescent tracer imaging in colon cancer: A systematic review and meta-analysis.* Crit Rev Oncol Hematol, 2021. **158**: p. 103149.
12. Codignola, C., et al., *Is there any role for sentinel node mapping in colorectal cancer staging? Personal experience and review of the literature.* Jpn J Clin Oncol, 2005. **35**(11): p. 645-50.
13. Emile, S.H., et al., *Sensitivity and specificity of indocyanine green near-infrared fluorescence imaging in detection of metastatic lymph nodes in colorectal cancer: Systematic review and meta-analysis.* J Surg Oncol, 2017. **116**(6): p. 730-740.
14. Qiao, L., *Sentinel lymph node mapping for metastasis detection in colorectal cancer: A systematic review and meta-analysis.* Revista Espanola de Enfermedades Digestivas, 2020. **112(9)**: p. 722-730.
15. Son, G.M., et al., *Multifunctional Indocyanine Green Applications for Fluorescence-Guided Laparoscopic Colorectal Surgery.* Annals of Coloproctology, 2021. **37**(3): p. 133-140.
16. van der Zaag, E.S., et al., *Systematic Review of Sentinel Lymph Node Mapping Procedure in Colorectal Cancer.* Annals of Surgical Oncology, 2012. **19**(11): p. 3449-3459.
17. Dahl, K., et al., *Identification of sentinel nodes in patients with colon cancer.* Eur J Surg Oncol, 2005. **31**(4): p. 381-5.
